# Artificial intelligence and robotics on the frontlines of the pandemic response: the regulatory models for technology adoption and the development of resilient organisations in smart cities

**DOI:** 10.1007/s12652-023-04556-2

**Published:** 2023-03-01

**Authors:** Cristiana Lauri, Fumio Shimpo, Maciej M. Sokołowski

**Affiliations:** 1 European University Institute, Fiesole, Italy; 2 Keio University, Tokyo, Japan; 3 University of Warsaw, Warsaw, Poland; 4 University of Macerata, Macerata, Italy

**Keywords:** Artificial Intelligence, Robotics, COVID-19 pandemic, Regulation, Smart Cities, Resilience

## Abstract

Smart cities do not exist without robotics and Artificial Intelligence (AI). As the case of the COVID-19 pandemic shows, they can assist in combating the novel coronavirus and its effects, and preventing its spread. However, their deployment necessitate the most secure, safe, and efficient use. The purpose of this article is to address the regulatory framework for AI and robotics in the context of developing resilient organisations in smart cities during the COVID-19 pandemic. The study provides regulatory insights necessary to re-examine the strategic management of technology creation, dissemination, and application in smart cities, in order to address the issues regarding the strategic management of innovation policies nationally, regionally, and worldwide. To meet these goals, the article analyses government materials, such as strategies, policies, legislation, reports, and literature. It also juxtaposes materials and case studies, with the help of expert knowledge. The authors emphasise the imminent need for coordinated strategies to regulate AI and robots designed for improving digital and smart public health services globally.

## Introduction

Smart cities do not exist without Artificial Intelligence (AI) and robotics. It is not only a matter of definition, because this correlation also has a practical aspect, as AI is becoming an increasingly important component of smart cities. This has been especially important during the COVID-19 pandemic, where AI and robots have been assisting in combating the novel coronavirus with its effects, and preventing its spread. However, as the experience of subsequent lockdowns and distancing measures demonstrates, the deployment of AI and robotics–as a matter of technological advancement and the level of technology available–must be tailored to the situation (pandemic), necessitating the most secure, safe, and efficient use of smart emerging technologies and AI.

With this in mind, the primary purpose of this article is to address the regulatory framework for AI and robotics in the context of developing resilient mechanisms in smart cities during the COVID-19 pandemic. The study provides regulatory insights to re-examine the strategic management of technology creation, dissemination, and application in smart cities, in order to address the issues regarding the strategic management of innovation policies nationally, regionally, and worldwide. This is particularly relevant to the deployment of AI solutions and the establishment of resilient organisations in smart cities to handle the regulatory challenges of the pandemic. To meet these goals, the article analyses a range of materials, such as strategies, policies, legislation, reports, and literature and also juxtaposes numerous case studies. This is done with the help of expert knowledge, enhanced by the activity of the *Global Pandemic Network* which brings together scholars from universities all over the world, in order to conduct research on legal, economic, and social issues related to pandemics (Benjamin et al. 2021).

The article seeks to expand the discussion on the governance of AI and robotics during the pandemic and provides an added value to the discussion on recent changes in the institutional environment. For these needs, the following research questions are discussed:

1. what are the contemporary legal issues underlying AI and robotics regulatory choices?
2. how have these issues translated into the use of AI and robotics in the fight against the COVID-19 pandemic?
3. what are the determinants for creating a regulatory model of AI and robotics for the needs of a development of resilient organisations in smart cities?

In this light, the structure of this paper is based on three pillars that showcase a discussion of a general regulatory framework for AI and robots, covering such issues as privacy and data protection (Sect. 2), case studies of AI and robots used to combat the COVID-19 pandemic (Sect. 3), and resilience regulatory modes based on public disaster management (Sect. 4). The final section of this study emphasises the main issues and provides some follow-up remarks.

## Development of policy approach for AI and robotics

Development of AI and robotics in smart cities requires an organised policy approach (see Tsuji 2018; Sokołowski 2022). This paradigm derives from the need to protect fundamental rights and freedoms, including the right to life, liberty and security of a person and privacy, or the right to freedom of opinion and expression (see Ufert 2020). In fact, the development of AI systems and robotics can profoundly undermine the value structure of some legal frameworks, especially in those where democratic constitutions guarantee a high level of protection of the rights and interests of an individual. Observing the pervasive capacity of such emerging technologies, legal analysis has developed along several lines. With reference to the introduction of systems designed to control people, conflicting with their freedom of movement and the protection of their personal sphere, regulators worldwide seek to strengthen the rules on privacy by default and design. Issues related to liability for harms caused by AI tools also require delineating the roles and individual responsibilities of the actors who use these tools, both in public and private activities. Moreover, algorithms can affect individuals’ freedom of choice, self-determination, and awareness, imposing the need for transparency rules to make users aware. The listed concerns are also sparking a debate on the distinction of roles between humans and machines that transcend law and touch the boundaries of ethics and philosophy, innovating the legal approach to be implemented in the future. This compels policymakers to take responsibility for providing the appropriate approach. What measures, however, should be implemented?

Depending on the socio-economic structure adopted, the degree of public approach inevitably varies, in case of technology policy as well (see Barge-Gil and Modrego-Rico 2008); however, two extreme points can be differentiated on the axis of this impact: total subordination and complete release, the total opposites (Sokołowski 2018a). These liberal and interventionist approaches, both rooted in the market- and state-based approaches (see Sokołowski 2016) are delimited by an intervening space filled by public law regulation (see Barnett 1986), a regulatory zone representing mixed economy (Sokołowski 2020a; see Harris 1990). The history of the development of such areas as telecommunications, energy, or aviation shows that the regulatory action is becoming the preferred approach (Sokołowski 2020a; Dempsey and Gesell 2013; Kolasa-Sokołowska 2022; Dempsey 1990). This tendency also applies to AI (see Scherer 2015; Clarke 2019), where the race to AI has also spurred a race towards AI regulation (Smuha 2021; see Chawla et al. 2022).

In contrast to the regulatory approach, one may find deregulation. It is the process of removing public components from a specific area, sector, or policy (Sokołowski and Heffron 2022). “For some, the problem will always be that the markets were not ‘free’ enough from government interference and a further reduction in regulation is needed” notices Thomas (2006, p 1975). This means that, in the most severe scenario, neither a public agency (regulator) nor the instruments of public regulation, such as control, commands, sanctions, etc., exist (Sokołowski 2018a, p 595). It can lead to a complete release in extreme cases, which, when paired with fraudulent behaviour and market manipulation (see Windolf 2004), can lead to a significant crisis (see Duane 2002; Sokołowski 2020a, pp 174–175).

A particularly appealing alternative to business is a policy that enables the avoidance of responsibility, e.g., by applying a soft regulatory approach – a “light touch regulation” (Fisk 2011, p 556) – which takes the risks of non-compliance by relying on the independent achievement of the set goals (Heffron et al. 2018, p 1193). While this approach may work well in the short term, for instance in a new technology development phase, or in the current COVID-19 pandemic (see Bachtiger et al. 2020), it necessitates a high level of trust in regulated entities – as the regulatory arrangement can be used by the regulated entity to relieve a burden of responsibility regarding duties – a regulatory capture (see Galloway 2020, pp 59–60). In the long run, it can result in misconducts, frequently discovered only in the final stages (Sokołowski and Heffron 2022). As a result, leaving the policy implementation in the hands of the regulated businesses (self-regulation) is not the best idea (see Lauri 2020), especially when no enforcement is available under the soft approach or the enforcement is of a very weak nature. Because the regulators’ and regulated parties’ interests differ in various ways (Bella et al. 2021), it is quite likely that this accommodative approach will fail (Sokołowski and Heffron 2022).

Nevertheless, there is a risk that during a pandemic, addressed under the state-of-emergency laws or some other extraordinary framework, overregulation characterised by the excessive creation of legislation and legal overweight will occur (Sokołowski 2018a, see 2020b, p 596). Cooperation can aid in preventing overregulation (see Sokołowski 2018a, p 596). In this improvement process, it will be useful not only to build a global data enforcement framework, based on the principles of privacy by design and privacy by default, but also to enforce basic principles for the upcoming regulatory assets. The said principles include transparency, interpretability, accountability, explicability, auditability, traceability, and neutrality or fairness (Bassan 2019; Kritikos 2020). The anticipated solution relates to the application of a global framework for the regulation of AI and robots, especially in order to define the conditions of their use, ensure the fairness of global competition, and conform their use to the protection of health and human rights according to the human centric approach (Lauri 2021b). Smart cities, with their smart infrastructures, are ideal testing grounds for implementing this approach (Cook et al. 2018; Obringer R, Nateghi R 2021). Table 1 summarises the benefits and drawbacks of the discussed policy approaches.

**Table 1:** Advantages and disadvantages of the selected policy approaches

Approach	Advantages	Disadvantages
State monopoly	Organisational subordination in accordance with state policy	Broad state command and control in a rigid, closed to competition centralised structure
Regulation	Balancing state and private interest to protect fundamental rights and freedoms	Quality of regulatory action based on adopted procedures as well as powers and resources attributed to regulator
Deregulation	Free competition and market openness	Risk of fraudulent behaviour and market manipulation
Soft regulation	Greater market flexibility with some elements of state control	Risk of non-compliance, especially in the final phase
Self-regulation	Rapid regulatory response and adaptation to changing circumstances	Risk of mismatching standards with the highest quality of protection

Let us refer this to issues such as privacy and data protection. When numerical statistics are collected and analysed with a help of AI or by AI itself, and then combined with other personal data, they become “personally identifiable information” (Shimpo 2020). This necessitates the development and implementation of appropriate approach, including legal, to protect the right to privacy (Florencio and Ramanathan 2001, p 105). This is particularly essential given that it is regarded as a universal human right (Blasi Casagran 2017, p 228) and considered basic in many jurisdictions around the world (see Parker 2010). Here, in setting the rules to deal with new problems, EU regulatory initiatives have been ahead of the curve in many respects, for instance in terms of tackling climate change (see Perez de las Heras 2013; Sokołowski 2018b). This also concerns the area of data protection (see Pajuste 2019), in which there has been a remarkable degree of legislative upgrading (in the EU), from the Data Protection Directive 95/46/EC (Data Protection Directive 1995) to the EU General Data Protection Regulation (GDPR) 2016/679 (GDPR 2016). The degree of transition and upgrading of the legislation is remarkable. In addition, the EU has been deliberating on an AI Regulation and is looking ahead to lead the debate on AI regulation in the future. Recently, the European Commission (2021a) proposed new rules regarding the promotion of regulation for trustworthy AI. The first-ever legal framework on AI, combined with the new Coordinated Plan of Member States, will ensure people’s and enterprises’ safety and basic rights, while also boosting AI use, investment, and innovation across the EU. Moreover, the proposed new EU rules on machinery (European Commission 2021b) will complement these efforts by adjusting the safety rules to boost users’ confidence in the next generation of products (European Commission 2021c).

Regulated regimes can help to mitigate the scope of these issues, but some concerns may still persist (see Dickinson et al. 2021). For instance, if a problem arises because of AI making an autonomous decision, such as taking the wrong action and causing damage, can we hold it liable under traditional product liability? Even if we can claim product liability for a robot equipped with AI, how should we view the fact that in the future AI might make decisions that we cannot predict? Even if we can sue for product liability for AI-enabled robots, how should we compensate for the damage caused by the AI programme itself running out of control or operating in an uncontrollable state? This is especially true when these robots are constantly connected to the network and used in the Internet of Things (IoT) applications daily; as a result, the problem will be present everywhere.

Because the IoT aims to influence the use of everyday objects via the Internet, we must consider legal issues when these objects are connected to robots, and the robots themselves are connected to the network and controlled by AI for use in our daily lives (autonomous robots). The issues surrounding autonomous robots equipped with AI, whether general-purpose or specialised, are expected to differ from those surrounding industrial robots. However, the primary legal issues surrounding robots continue to be those pertaining to industrial robots. Furthermore, some believe that existing robot safety standards will be adequate to deal with any new issues that arise. Let us now examine these issues through the lens of the COVID-19 pandemic’s use of robots and AI.

## AI and robots in the fight with COVID-19: governance issues

In 2018, the World Health Assembly Resolution on Digital Health recognized the value of digital technologies to reach the Sustainable Development Goals (see WHO 2019; WHO 2021). In 2020, as the COVID-19 pandemic spread worldwide, several forms of technological applications started to be massively implemented. Among them Kritikos (2020) lists: AI, blockchain, open-source technologies, telehealth technologies, three-dimensional printing, gene-editing technologies, nanotechnology, synthetic biology, and, lastly, drones and robots. Without disparaging the initial elements of this list, the final few have a wide range of applications in fighting the novel coronavirus. For instance, according to the *Robotics for Infectious Diseases* consortium, more than 150 robots are being used to combat COVID-19 (Vargo et al. 2021).

Indeed, during the pandemic, digital technologies have been widely introduced in a number of areas of intervention that characterise smart cities (Pacheco Rocha et al. 2019; Lauri 2021b) and their intrinsic purpose of improving the quality of life in densely populated urban contexts (see Lytras et al. 2019b). This can be summarised as follows (Murphy et al. 2020, 2021): public safety (for compulsive quarantine enforcement, disinfection of public spaces, identification of infected individuals, public service announcements, and traffic flow monitoring); clinical care (for point-of-care disinfection, observational telepresence, delivery and inventory, interventional telepresence, patient and family socialization, and patient and visitor admissions); continuity of work and education (for sanitisation at work or school, for telepresence, for private health surveillance and security); laboratory and supply chain automation (for delivery activities, laboratory automation, management of infection materials); quality of life (delivery of food and other purchases, attending social events, interpersonal socialization); and non-hospital care (for delivery to quarantine, socialisation in quarantine, and public health surveillance). Table 2 summarises the technological applications used during the COVID-19 pandemic in relation to the public interest to be fulfilled, considering their type of use and the specific activities performed (Murphy et al. 2020, 2021).

**Table 2:** Technological applications and their use during the COVID-19 pandemic

Technological Applications	Public interests	Type of use	Activities
AI Blockchain Open-source technologies telehealth technologies Three-dimensional printing Gene-editing technologies Nanotechnology Synthetic biology Drones Robots	Health systems resilience	Public safety	Quarantine enforcement Disinfection of public spaces Identification of infected people Public service announcements Traffic flow monitoring
		Clinical care	Point of care disinfection Observational telepresence Delivery and inventory Interventional telepresence Patient and family socialization Patient and visitors admissions
		Non-hospital care	Delivery to quarantined Quarantine socializing Public health surveillance Off-site testing
	Resilience of work and social activities	Work and education	Sanitation of work/school Telepresence Process automation construction and agriculture Private health surveillance Private security
		Laboratory and supply chain automation	Delivery Laboratory automation manifacture Infectious material handling
		Quality of life	Delivery food and purchases Attending public social events Interpersonal socializing

Early detection and infection monitoring by AI and robots, in particular, have been crucial in the fight against COVID-19 (Pazzaglia et al. 2021); however, this activity has aroused concerns about the compatibility with legal compliance. Thus, it is no coincidence that most legal studies have been concerned with studying their utility for public health, considering issues of efficacy, equity, and privacy (Landau 2021). While the collection of real-time public health data has been advantageously implemented in both assisting the policymakers in the planning process and informing the public about the evolution of the pandemic’s spread (Budd et al. 2020), the governments’ access to various data, including a person’s geolocation, has raised numerous privacy issues (see Gerke et al. 2020; Hassandoust et al. 2021; Chan and Saqib 2021). These applications are not limited to a single country (see Chakraborty et al. 2020). For example, since October 2020, the EU ensures interoperability of COVID-19 contact tracing and warning apps in order to facilitate free movement as an integral part of the Single Market (European Commission 2020a).

Nevertheless, during the peak months of 2020 and 2021 globally, different models emerged for approaching the tracking activity. One is the *Chinese* model, while the other is the *European* model. Several different approaches can be found in the Asian context. Considering China, parallel with the outbreak of the contagion the Chinese authorities started to lay down *strong* measures to track the movement of people who had visited the Wuhan market. It was done through tools such as mobile phones, mobile payment applications, social real-time data on people’s location (Whitelaw et al. 2020) and facial recognition. This also allowed the authorities to forecast the transmission of the virus and orient border checks and surveillance strategies. For example, China used the *AliPay HealthCode* app for automatic communication and the enforcement of quarantine measures by limiting transactions permitted for high-risk users (Kupferschmidt and Cohen 2020).

While this system has enabled a drastic containment of the pandemic, the need to increase data protection is a topic of growing interest in Chinese law (Greenleaf 2020). The initial response began to emerge in October 2020, when parliamentarians began debating the Personal Information Protection Law (PIPL) to regulate the collection and use of personal data. In August 2021 the law was adopted, and will go into effect at the beginning of November 2021. PIPL is also intended to enhance the exchange of data with countries that have a higher level of protection and do not tolerate collection systems with no users’ consent or through non-transparent processes. In particular, Article 49 of the law stipulates that “[p]ersonal information handlers shall establish mechanisms to accept and handle applications from individuals to exercise their rights. Where they reject individuals’ requests to exercise their rights, they shall explain the reason”. The above mentioned “mechanisms” proceed in the direction of the European-derived legal meaning of privacy by design and privacy by default (Bifulco 2018). More specifically, such mechanisms would seem to recall those automatisms referred to by the GDPR (Article 25) that should allow data controllers (and consequently data processors) to carry out a processing operation by providing, from the outset (by design), the tools and correct settings to protect personal data, so that the framework of principles is respected by default. The PILP, along with the Data Security Law (implemented from since September 2021) mark two major regulations set to govern China’s smart cities in the coming years. These rules will affect the big tech companies who are the main actors in smart cities, as the new law will change the value of data and have a significant impact on their business and relations with institutions.

Moreover, countries such as South Korea have integrated AI and robotics into the government-coordinated containment and mitigation processes for early disease detection. These include surveillance, testing, contact tracing, and strict quarantine, even using geolocalisation and video surveillance measures (Zastrow 2020). South Korea, in other terms, has adopted a soft policy of voluntary containment, with widespread dissemination of information to citizens. The system is based on the central government’s existing smart city project, and is being developed in consultation between various ministries and the Centres for Disease Control and Prevention. The Korean system enjoyed a higher level of resilience when compared to others. And indeed, Korean law, amended after the 2015 MERS outbreak, provides a specific legal basis to allow authorities to access camera data, GPS tracking data from phones and cars, credit card transactions and other personal data for infectious disease control purposes. Access to this data by health professionals must still be authorised by law enforcement authorities, but the most recent changes (as of March 2020) allow also direct access by health authorities. The real time data and monitoring can support administration in management of smart services and control for better governance (see Kumar et al. 2020b). A public service enhanced by AI allows for community interaction that is tailored to the end users’ perceptions and abilities, and promotes individuals’ involvement in the community (Lytras et al. 2021). This is especially critical when dealing with the pandemic, which necessitates cohesion, collaboration, and coordination. Thus, the data that flows to the authorities not only supports the government’s (both central and local) activities in combating the spread of the coronavirus, but also keeps the population constantly informed – by the authorities – of these activities, and of the spread of the contagion. The secured data flow represents a tool for cooperation between the authorities and citizens, which can also help to maintain a balance between lockdown rules and normal life in times of high social tension.

Based on the experience of South Korea, some European states have begun to design a soft control system, that included: controlling quarantined persons through geolocation; tracking the routes of infected persons to identify those at risk; disseminating information to the public on the movements of infected persons to alert those at risk and invite them to undergo diagnostic tests. Such an approach has made it possible to comply with the requirement of “proportionality” between data protection and the interests of individuals (as highlighted in GDPR). As emphasised in the European Data Protection Supervisor’s guidelines (European Data Protection Supervisor 2020), compliance with the GDPR’s regulatory framework on privacy does not allow for strong pervasiveness of technological tools in the EU. For example, data collection in Norway through the *Smittestopp* app has been stopped due to its “disproportion to the task” (Budd et al. 2020). Therefore, it should come as no surprise that one of the envisaged solutions has been the implementation of *soft* apps, such as the Italian one called *Immuni.* The app is based on technical requirements aimed at balancing privacy and personal rights (De Falco and Maddalena 2020) with the detection action carried out with the support of an algorithm, thanks to the use of Bluetooth technology (European Commission 2020b). The soft approach is based on the following assumptions: first, the freedom of the user to download the app or not (without prejudice to those who evade); second, the transparency towards the subject regarding the use that is made of the users’ data; third, the determinacy and exclusivity of the data as far as statistical or scientific aims are concerned; fourth, data storage on a governmental server, for the duration of the pandemic; fifth, the reciprocity of anonymity, as citizens are limited to receiving a notice only in the event of interaction with an infected person; and finally, the selectivity, the minimisation of data and its pseudonymisation (Article 26 of the GDPR) according to the decentralised PEPPT (Pan-European Privacy Preserving Proximity Tracing initiative, 2020) model (Bonomi 2020).

As previously observed, AI and robotics have even been used to prevent rule violations in order to stop the spread of COVID-19, such as general lockdown and quarantine for people exposed to or infected with the virus. Even in legal systems with highly developed privacy-protection regulations, governments have used telephone traffic data obtained from internet providers (data retention) to tackle the pandemic (Oliver et al. 2020), given the need to repress behaviours capable of undermining public health. Only data pseudonymisation and anonymisation could alleviate privacy concerns in this scenario.

Prevention and monitoring activities are the prerequisite for stabilising the *new situation*, but a further step in building a resilient system is bolstering healthcare services. This is especially important in metropolises and urban complexes due to human congestion. As a result, contact tracking applications may aid in the development of smart cities, benefiting public transportation and related industries while also providing valuable insights for city management (see Schmidtke 2020, p 200). The definition of a clear framework in terms of privacy by design and by default constitutes the basis for introducing different AI and robotics systems useful for enhancing smart cities and improving citizens’ well-being. Table 3 summarises the various approaches used by different legal systems to track the activity of infected individuals, considering the technology involved, the main objective pursued by legislators, and highlighting advantages and disadvantages.

**Table 3:** Approaches to track the activity of infected individuals

Area	Approach	Technology	Main objective	Advantages	Disadvantages
China	Strong	Tracking of mobile phones; mobile payment applications; social real-time data; facial recognition	Forecasting the transmission of the virus Orienting border checks and surveillance strategies	Rapid containment of the pandemic spread	Lack of privacy and data protection framework
South Korea	Soft	Contact tracing; geolocalisation; video surveillance measures	Supporting public administration through the integration of AI and robotics into the government-coordinated containment and mitigation processes	Keeping the population constantly informed	Need for enforcement of privacy and data protection framework
EU	Soft	Interoperability of contact tracing and warning apps	Facilitating free movement in the EU	Voluntary system; high level of privacy protection	Risk of lack of effectiveness; need for manual contact tracing

Yet another facet of healthcare and the use of AI in the COVID-19 pandemic is the utilisation of robots for assisting healthcare workers. For instance, disinfection of spaces in public buildings such as schools and hospitals, but also delivering food and medical supplies (see Bogue 2020). Using robots in medical activities has a positive impact on improving the smart city’s health resilience. The rapid use of IoT devices has facilitated the collection of health-related big data (see Lytras et al. 2019a). Many medical facilities have begun to fully digitise electronic health records for clinician testing orders, referrals, and patient scheduling in order to improve the efficacy and efficiency of both medical and administrative healthcare processes (see Flynn et al. 2020). Deep learning has been used to diagnose COVID-19 using X-ray pictures (Wang et al. 2020). AI can be used to track the spread of COVID-19 and predict a patient’s needs. Through computational biology and the use of data analytics, mathematical modelling and computational simulation have helped to study and research the pandemic (Kumar et al. 2020a).

Furthermore, many medical facilities have started to introduce robots in therapies. Such is the case of Loccioni, a company which used a robot to prepare a monoclonal to treat COVID-19 patients at the Hospital of Ancona (Italy). Robots can autonomously carry out the most complex operations in order to guarantee the correct composition of the therapy and intercept any possible errors during validation, transcription, preparation and delivery. Based on physicians’ reports, eligible patients are received in special rooms set up at the infectious diseases department, in a protected environment with negative pressure. The therapy requires utmost care and precision during the drug preparation procedure. The personalised preparation of injectable drugs represents a critical aspect for healthcare facilities as it involves numerous risks for the safety of patients and operators, as well as significant costs and possible organisational inefficiencies. The entire drug pathway, from prescription to administration, is controlled through sophisticated automated measurement systems that ensure high accuracy, complete traceability of operations and integrity of information. The prescription is digitised and the preparation phase takes place in a fully automated manner, in a dedicated and constantly monitored work environment. As a result, these therapies are confirmed by quality certificates, offer maximum safety in terms of sterility and accuracy of the injectable drugs prepared, allow safe management of clinical data and the production phase, and reduce clinical risk (Yaniv et al. 2017).

Indeed, since the spread of COVID-19, previously harmless tasks may pose serious health risks. In places too dangerous for them, humans are being replaced by robots, which are considered more reliable and cost-effective. However, any advantages in terms of health risk prevention are matched by the risk of job losses for all those whose tasks are going to be taken over by technologies (Ramirez 2021). “Retraining unemployed people was never easy, but it is more challenging now that technological disruption is spreading so rapidly, widely, and unpredictably” highlights Floridi (2017, p 3). This fact is linked to a broader reflection on the loss of humanity in certain activities and relationships, which, along with issues of privacy and security, is part of the debate that many legal systems are facing in preparing a regulatory framework for the use of AI (Bassan 2019).

The application of AI and robots, discussed here, is a tool for strengthening the resilience of the public health service on several fronts (Auby 2020). Moreover, it offers at least three methodological considerations helpful for understanding the coordinates on which to develop the regulatory framework. First, the public–private partnership created to develop the robots between the public (in this case, the hospital) and the private company combines the expertise of the national service system and the know-how of the private company. This promotes an increase in the organisational efficiency and ergonomics of the process (Valaguzza and Parisi 2020). It also makes it possible to move away from the dependence of the service on public resources, which are often insufficient, and to be able to rely on the economic investments and resources of the private sector. The adopted method of shared governance also allows the reengineering of processes through the sharing of best practices, in order to bring innovation to the public sector, considered extremely conservative both for the scarcity of resources available and still underdeveloped culture of innovation. Second, AI and robot introduction in ordinary medical activities prevents human errors and accurately controls the appropriateness of the medical prescription. This is a form of “preventive medicine actions” and can be useful to create “personalized services”, adaptable to the patient and highly efficient, as recommended by the European Communication on Digital Health Services (2018). Third, the implementation of robotics simplifies documentation management by making information more usable for the benefit of the patient. There is thus an advantage in terms of transparency of the service provided and of knowability, creating a more collaborative environment and reinforcing trust between treatment facilities and patients.

## AI, robots, and smart cities: COVID-19 resilience regulatory model

A re-examination of the strategic management of technology creation, dissemination, and application in smart cities is required to build resilient organisations in smart cities during the COVID-19 pandemic with the help of robotics and AI (de Pablos et al. 2022). Disaster management provided by public authorities when dealing with the effects of hurricanes, earthquakes, or tsunamis constitutes a useful benchmark (Sokołowski 2020b). This refers to: the application of AI and robotics to enhance the public’s ability to respond to disasters, policymaking under unusual circumstances (see Schneider 1992), or remedies (ex-post disaster assistance or ex-ante regulation) to limit loss exposure (see Priest 1996, p 219) or alleviate disaster effects (Malawani et al. 2020). A pandemic, if treated as a calamity that may reoccur – like in the case of SARS-COV-3, SARS-COV-4, or any other infectious disease – makes preparedness the key element of regulatory approach (see WHO 2019), and a critical component of true smart cities, which are primarily targeted by the current pandemic’s negative effects (being large clusters of people). As a result, it is necessary to consider the future challenges now, while, at the same time, continuing to implement the measures aimed at combating the current pandemic. In this regard, considering a pandemic as a natural disaster that may reoccur demonstrates the validity of referring to a regime designed to counteract natural disasters (see Sokołowski 2020b; Dixit 2020; Tsuji 2021).

Furthermore, many parallels can be found in the current pandemic between activities related to those undertaken by public authorities. For example, following Hurricanes Katrina and Rita in 2005, federal regulatory agencies recognised that, due to extraordinary circumstances, flexibility in the application of rules and simplifying several applications were required (Sokołowski 2020b). This also concerns certain regulatory reliefs offered to professionals vital in disaster response or recovery, e.g. by adjusting licensing requirements, or freezing inspections (Sokołowski 2020b). AI and robotics offer a wide range of possibilities in this area. This is about simplifying procedures, making them more responsive as well as contactless, and conducting them online. Innovative chatbots can offer a straightforward support in administrative procedures, for example, when applying for licences or certificates (see van Noordt and Misuraca 2019), while different AI applications can perform inspections comparable to those carried out by humans (for instance, a drone – an unmanned aerial vehicle equipped with a camera conducts technical monitoring of a power line). However, this requires a regulatory environment that recognises the equivalence of such activities to those carried out in a traditional manner. Smart cities are the perfect environment for introducing such improvements.

Furthermore, as in disaster prevention, AI offers enormous modelling possibilities, providing expert forecasts on pandemic development. These models can be utilised on a voluntary basis; however, a legal approach should guide their development. This could be done, for example, by offering specifications for their use (including the scope of the analysed data), as well as listing institutions that should use them (for instance, by making it mandatory for health establishments). This is also a source of concern for city authorities, particularly those in metropolitan areas, as the health-care administration (naturally at the forefront) is not the only one working to combat the pandemic. Other institutions are also striving to ensure compliance with the law and standards, as well as transparency and clarity of rules regarding consumers and competition in extraordinary times (Sokołowski 2020b).

Moreover, fighting the pandemic demonstrates the importance of well-functioning coordination systems; coordinating policies can improve the effectiveness of crisis response (OECD 2020, p 2). This is especially true in those circumstances when the central government plays a larger role – worldwide examples of actions performed during the COVID-19 pandemic illustrate that this involvement frequently overshadows activities of other entities, e.g., local authorities (Sokołowski 2020b). In such situations, AI technologies can improve the coordination mechanism of a multi-actor administration system, making it more effective and responsive. The widespread adoption of AI should be a post-pandemic standard, transforming traditional administration into true e-administration (see Wierzbowski et al. 2021). The same applies to the transformation of traditional cities into smart ones (see Bobadilla et al. 2018).

This also concerns the structure of administration, both central and local. If – apart from coordination mechanisms – a specialised anti-pandemic authority is established (for example, an agency responsible for combating COVID-19), it can, in addition to all necessary expert knowledge obtained from the health administration, serve as a valuable benchmark of an e-administration scenario. In such conditions, AI applications can not only help with the creation of a structure solely responsible for countering COVID-19 (or future, similar events), but also accelerate the process of transformation to e-administration at different levels (van Noordt and Misuraca 2019). This, of course, also applies on a city level, as no real smart city can exist without e-administration (Lauri 2021a).

**Fig. 1:**
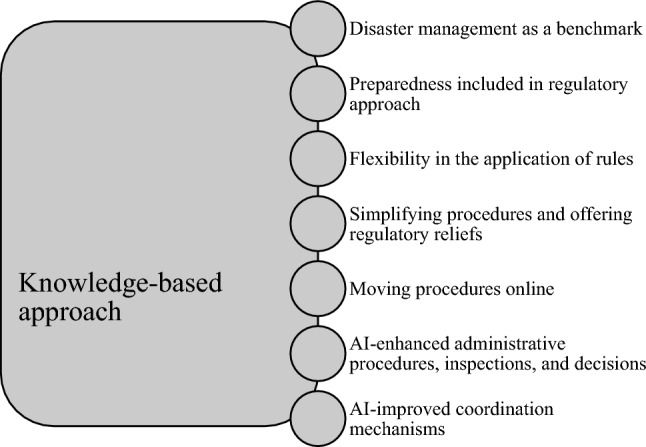
Key elements of the COVID-19 resilience regulatory model

Finally, AI has a wide range of applications connected to the knowledge-based approach (see Fig. 1), which could result in adopting a system of rules, standards, authorisation, permissions, and guidance dedicated to COVID-19, based on best available practices as much as feasible (see Sokołowski 2020b). These should be accompanied by pandemic-specific monitoring, surveillance, and enforcement that is as safe (non-physical, online, etc.) as possible, free of unnecessary administrative hassle and with deadlines suspended or extended (Sokołowski 2020b). Of course, it must be scaled to the challenge – the recent COVID-19 variants, especially the quickly spreading omicron, make it far more difficult to adjust state logistics to the size of the problem (as is the case, for example, in South Korea). With such an approach, AI can help authorities, also in cities, become smarter and more resilient organisations, guided by pragmatic and responsive regulation. Nevertheless, this process requires some universal standards. The urgent need for coordinated, global, digital and smart public health strategies has been highlighted both by the WHO, in its global strategy on digital health 2020–2025 (WHO 2021) and by the EU, which called for a pan-European approach on the use of data for COVID-19 (European Commission 2020a, b) currently also being implemented through collaborative research projects (Tacconelli et al. 2022). Moreover, coordination serves as an ancillary element to bridge the digital divide by ensuring access to mobile communication and internet services, particularly in low- and middle-income countries, as well as for minorities and people with lower socioeconomic status. Indeed, an unequal access to technology can exacerbate inequalities between countries in terms of their preparedness to fight future pandemics, which can jeopardise the resilience of all the areas of the world. This also concerns cities, as the smart ones are at the forefront, while the “analogue” ones are lagging behind (Thomas et al. 2021).

## Conclusion

The rapid advancement of science and technology, the increased use of information technology, and the development of network-related technologies have all resulted in significant improvements to our daily lives, opening several legal issues. This also applies to governance of robotics and AI that have also helped profoundly in the frontline fight against COVID-19 in the urban environments. These actions, however, have frequently caused or exacerbated legal issues related to the employment of AI and robotics.

As discussed in this study, looking at issues in an institutional system AI solutions can improve the coordination mechanism of a multi-actor administration system, making it more effective and responsive. For instance, private public partnerships combine the know-how of the private enterprise with the knowledge of the national service system, optimising the possibilities of delivering the best results in terms of preventing human errors, appropriateness of medical actions, and ease of documentation administration. However, as these new and emerging technologies have been introduced into societies and cities and their use has increased, certain problems have arisen – not only from illegal activities or misuse (that should be regulated by law), but also from the lack of rules governing the use of the said technologies. This applies not only to surveillance systems aimed at preventing the spread of a pandemic, which – as can be seen – may follow “soft” or “strong” approaches depending on regulatory frameworks; it refers also to the digitisation of services (as in the use of robotics for health services).

Given this scenario, what are the determinants for creating a regulatory model of AI and robotics for the needs of a development of resilient organisations in smart cities? As identified in the paper, among current legal issue there is a need for a responsive regulatory framework of a universal character (at least at a level of principles, as due to the diversity of legal systems, it is challenging to attain complete universality of solutions at the global level), which can simultaneously hold together the protection of privacy and the rights of individuals and the fulfilment of public interests. Indeed, as it turns out, with AI and modern technologies consistent with a regulatory system, it is possible to improve the resilience of health systems and work and social activities, which are essential prerequisites for contextualising smart cities in an institutional system. This calls for smart regulation, driven by knowledge-based approach, with disaster management as a benchmark and preparedness included in regulatory approach, bringing flexibility in the application of rules, simplifying procedures and offering regulatory reliefs, and moving procedures online, with AI-enhanced administrative procedures, inspections, and decisions, and last but not least AI-improved coordination mechanisms.
